# Unresolved Excess Accumulation of Myelin-Derived Cholesterol Contributes to Scar Formation after Spinal Cord Injury

**DOI:** 10.34133/research.0135

**Published:** 2023-05-04

**Authors:** Bolin Zheng, Yijing He, Shuai Yin, Xu Zhu, Qing Zhao, Huiyi Yang, Zhaojie Wang, Rongrong Zhu, Liming Cheng

**Affiliations:** ^1^ Key Laboratory of Spine and Spinal Cord Injury Repair and Regeneration, Ministry of Education, Department of Orthopedics, Tongji Hospital, School of Medicine, Tongji University, Shanghai 200092, China.; ^2^ Frontier Science Center for Stem Cell Research, School of Life Science and Technology, Tongji University, Shanghai 200092, China.; ^3^ Clinical Center for Brain and Spinal Cord Research, Tongji University, Shanghai 200092, China.

## Abstract

Spinal cord injury triggers complex pathological cascades, resulting in destructive tissue damage and incomplete tissue repair. Scar formation is generally considered a barrier for regeneration in the central nervous system. However, the intrinsic mechanism of scar formation after spinal cord injury has not been fully elucidated. Here, we report that excess cholesterol accumulates in phagocytes and is inefficiently removed from spinal cord lesions in young adult mice. Interestingly, we observed that excessive cholesterol also accumulates in injured peripheral nerves but is subsequently removed by reverse cholesterol transport. Meanwhile, preventing reverse cholesterol transport leads to macrophage accumulation and fibrosis in injured peripheral nerves. Furthermore, the neonatal mouse spinal cord lesions are devoid of myelin-derived lipids and can heal without excess cholesterol accumulation. We found that transplantation of myelin into neonatal lesions disrupts healing with excessive cholesterol accumulation, persistent macrophage activation, and fibrosis. Myelin internalization suppresses macrophage apoptosis mediated by CD5L expression, indicating that myelin-derived cholesterol plays a critical role in impaired wound healing. Taken together, our data suggest that the central nervous system lacks an efficient approach for cholesterol clearance, resulting in excessive accumulation of myelin-derived cholesterol, thereby inducing scar formation after injury.

## Introduction

Spinal cord injury triggers complex pathological cascades [1], which result in destructive tissue damage and culminate in incomplete tissue repair characterized by scar formation [2,3]. A spinal cord scar generally consists of a glial scar border, also known as an astrocyte scar, and a non-neural lesion core, also known as a fibrotic scar [2]. Astrocyte scars have been proposed as barriers to axonal regrowth, but recent evidence has indicated that astrocyte scar formation can be beneficial for tissue repair and axon regeneration [4–6]. Fibrotic scars, primarily composed of fibroblasts and bone marrow-derived macrophages (BMDMs) [7], impede tissue regeneration [8–10]. Notably, in contrast to the sustained accumulation of activated macrophages/microglia in adult lesions [7,11], transient activation of microglia mediates glial bridge formation and organizes scar-free healing in neonatal mice [12], similar to that in zebrafish and newts [13,14]. While the role of spinal cord scars has been well demonstrated [10], the intrinsic mechanisms that lead to scar formation are largely unknown.

Cholesterol is an indispensable constituent of the mammalian biological membranes. However, excess cholesterol accumulation induces ubiquitous toxicity that is involved in the progression of numerous diseases [15]. These diseases include atherosclerosis, nonalcoholic fatty liver disease, chronic kidney disease, diabetes, immune dysfunction, coronavirus disease 2019, and Alzheimer’s disease [15]. Several mechanisms can protect cells from excess cholesterol accumulation [16]. Preferentially, excess free cholesterol is esterified into cholesteryl esters for storage in lipid droplets. Meanwhile, cellular cholesterol is exported and delivered through reverse cholesterol transport (RCT) from peripheral tissues to the liver [16]. Under certain conditions, ineffective cholesterol efflux results in foam cell formation loaded with esterified cholesterol, free cholesterol, and crystallized cholesterol owing to the hydrophobic property [17,18].

The central nervous system (CNS) contains more than 20% whole-body cholesterol, up to 70% of which resides in the myelin [19]. Following spinal cord injury, a large amount of cellular and myelin debris is generated and then largely engulfed by BMDMs [20]. Although myelin debris is well known to not only inhibit axon regeneration but also mediate inflammation [21,22], the process and consequence of myelin degradation in phagocytes remain elusive. After spinal cord injury, abundant lipid-laden macrophages are present in the lesion core [20]. Moreover, the transcriptional profiles of these lipid-laden macrophages closely resemble foam cells at 7 d post injury (dpi) and are predominantly enriched in lipid catabolism in gene ontology (GO) analysis [23]. These findings indicate that lipids, degradation products of cellular and myelin debris, are involved in the pathological process of spinal cord injury. Considering that a large proportion of cholesterol resides in the CNS and that excess cholesterol plays a crucial role in multiple diseases, elucidating cholesterol metabolism and its potential consequences in CNS lesions is essential.

This study highlights the role of cholesterol homeostasis in spinal cord lesions. Using confocal reflection microscopy, we detected cholesterol crystals in spinal cord lesions as early as 7 dpi, which mediates inflammasome activation. We found that excess cholesterol also accumulates in injured peripheral nerves but is subsequently removed by RCT. Moreover, prevention of cholesterol efflux after peripheral nerve injury results in macrophage accumulation and fibrosis. We further demonstrated that myelin-derived cholesterol is sufficient for excess cholesterol accumulation in spinal cord lesions, which contributes to persistent macrophage activation and fibrosis.

## Results

### Cholesterol crystals are present in the spinal cord lesions of young adult mice

**Fig. 1.**
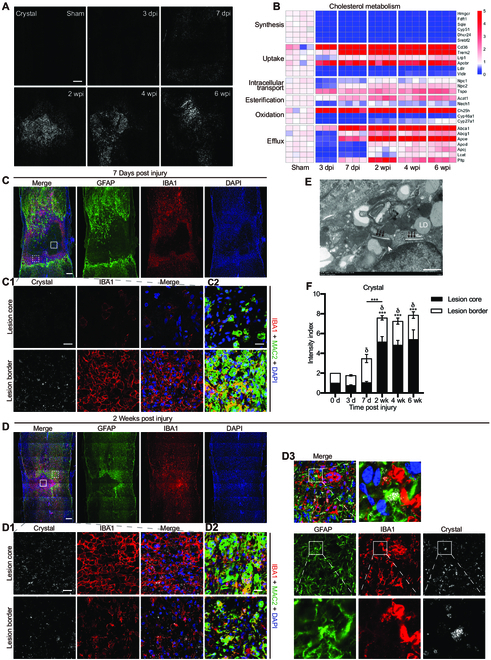
Accumulation and distribution of cholesterol crystals in spinal cord lesions. (A) Representative reflection images of spinal cord lesions in young adult mice taken at different time points after injury. The reflection signal is shown as white. (B) Quantification of the time-course expression of cholesterol metabolism-related genes in spinal cord lesions using real-time PCR. (C and D) Confocal images of spinal cord lesions stained with GFAP (green), IBA1 (red), and DAPI (blue) taken at 7 dpi and 2 wpi, respectively. (C1 and D1) Confocal fluorescence and reflection images from boxed areas (box, lesion core; dashed box, lesion border) in (C) and (D), showing crystals (white) in IBA1-positive phagocytes (red). (C2 and D2) Representative images of the lesion core and lesion border stained with IBA1 (red), MAC2 (green), and DAPI (blue) at 7 dpi and 2 wpi, respectively. (D3) Representative image of the lesion border stained with GFAP (green), IBA1 (red), and DAPI (blue) taken at 2 wpi, showing crystals (white) in GFAP-positive astrocytes (green). The boxed areas are shown magnified. (E) Representative transmission electron microscopy image of a spinal cord lesion showing needle-like cholesterol crystals (black arrows) and lysosomes (white arrowhead) taken at 2 wpi. LD, lipid droplet. (F) Quantification of the crystal intensity (normalized to the proximal region) in the lesion core and lesion border (*n* = 4 mice). Ordinary 1-way ANOVA with Tukey’s multiple comparisons test. Data are shown as means ± SEM. ***P < 0.001 (lesion core: each column vs. the column of Sham unless indicated). ^δ^*P* < 0.05 (lesion border: each column vs. the column of Sham). Scale bars: 200 μm (A, C, and D), 20 μm (C1, C2, D1, D2, and D3), and 1 μm (E).

To investigate the specific role of lipids in pathological processes after spinal cord injury, we focused on cholesterol homeostasis because cholesterol is extremely rich in the CNS. Using confocal reflection microscopy [17], we detected a large number of cholesterol crystals, a typical hallmark of excess cholesterol accumulation, as early as 7 dpi in the spinal cord lesions of young adult mice (Fig. 1A). Cholesterol crystals increased at 2 weeks post injury (wpi) and accumulated in the lesions for at least for 6 weeks (Fig. 1A and F). We verified that needle-like cholesterol crystals in suspension and multidirectional cholesterol crystals in the mixed suspension of crystal suspension with Fluoromount-G mounting medium were detected using confocal reflectance microscopy; however, no signals were detected in adipose tissues (Fig. S1C). Combined with confocal fluorescence microscopy, we detected crystals deposited in ionized calcium binding adaptor molecule 1 (IBA1)-positive phagocytes at 7 dpi (Fig. 1C and C1) but not at 3 dpi, when BMDMs initially infiltrated into the lesion. Along with macrophage/microglia centripetal migration, the scar was sealed and matured at 2 wpi [24,25] (Fig. 1D). In the lesion core of the mature scar, crystals were primarily present in macrophages (Fig. 1D1). At the lesion border of the mature scar, crystals were present in both the macrophages and astrocytes (Fig. 1D1 and D3 and Fig. S1B). In addition, crystal-deposited phagocytes were strongly positive for macrophage-2 (MAC2) (Fig. 1C2 and D2 and Fig. S1A), suggesting that they are BMDMs [20]. Using transmission electron microscopy, the presence of cholesterol crystals was further verified, as needle-like crystals were observed in both the lysosome and cytoplasm of foamy macrophages (Fig. 1E). Tiny crystals were also present in the spinal cord lesions of rat (Fig. S1D). Our results suggest that phagocytes, particularly macrophages, are challenged by excess cholesterol and accumulate in spinal cord lesions.

Subsequently, we quantified the time-course expression of the cholesterol metabolism-related genes after spinal cord injury using real-time polymerase chain reaction (PCR) (Fig. 1B). On the one hand, injuries resulted in reduced expression of the genes related to cholesterol synthesis. By contrast, the injured spinal cord increased the expression of the genes involved in cholesterol uptake, including *Cd36*, *Trem2*, and *Apobr*, and cholesterol efflux, notably *Abca1* and *Apoe*. Meanwhile, the expression of *Acat1*, which mediates cholesterol esterification, and *Ch25h*, which mediates cholesterol oxidation, was also increased. Our results indicated that injured tissue is processing overloads of cholesterol derived from tissue debris in response to tissue damage.

### Excess cholesterol accumulation is associated with activation of NLRP3 inflammasome in spinal cord lesions

**Fig. 2.**
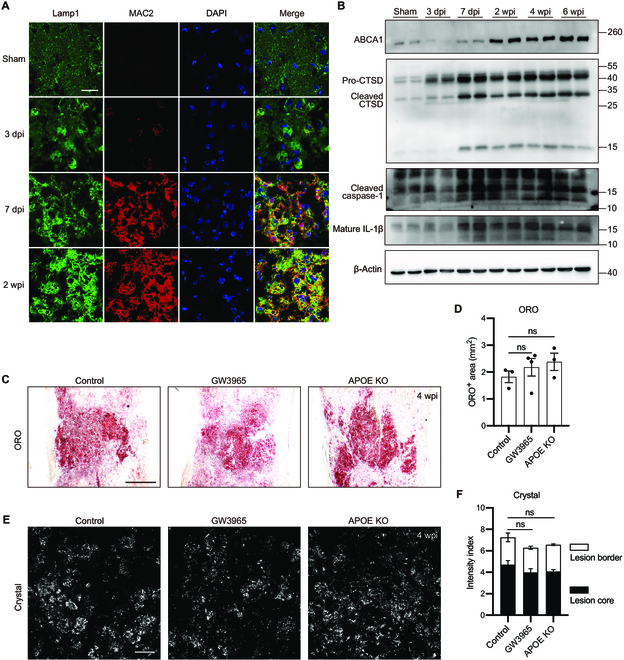
Unresolved excess cholesterol deposition and inflammation in spinal cord lesions. (A) Representative images of spinal cord lesions stained with LAMP1 (green), MAC2 (red), and DAPI (blue) at different time points after injury. (B) Immunoblots of spinal cord lesions for ABCA1, CTSD, caspase-1, IL-1β, and β-actin (loading control) at different time points after injury. (C) Representative images of spinal cord lesions in different groups stained with ORO (red) taken at 4 wpi. (D) Quantification of the ORO-positive area in (D) (*n* = 3, 4, and 3 mice for Control, GW3965 and APOE KO, respectively). (E) Reflection images of lesion cores in different groups showing crystals (white) taken at 4 wpi. (F) Quantification of the crystal intensity (normalized to the proximal region) in (F) (*n* = 3 mice). (D and F) Ordinary 1-way ANOVA with Tukey’s multiple comparisons test. All data are shown as mean ± SEM. ns, not significant. Scale bars: 20 μm (A and E) and 200 μm (C).

Crystal deposits induce various clinical disorders with shared molecular pathological mechanisms, among which NLR family pyrin domain containing 3 (NLRP3) inflammasome activation is known to trigger inflammation [26]. In atherogenesis, the formation of cholesterol crystals drives NLRP3 inflammasome-mediated inflammation by inducing lysosomal damage [17]. Moreover, in vitro phagocytosis of myelin debris leads to cholesterol crystallization and NLRP3 inflammasome activation [18]. Based on these findings, we examined inflammasome-mediated caspase-1 activation, which promotes the secretion of the proinflammatory cytokine interleukin 1β (IL-1β), in spinal cord lesions. At 7 dpi and 2 wpi, cholesterol-deposited macrophages showed colocalization of lysosomal associated membrane protein 1 (LAMP1), a marker of lysosomes, and MAC2/galectin-3, a marker of lysosomal membrane permeabilization [27] (Fig. 2A), suggesting that lysosomal membranes were ruptured. In addition, cathepsin D (CTSD), a lysosomal protease, increased markedly from 7 dpi. Both cleaved caspase-1 and mature IL-1β persistently elevated from 7 dpi (Fig. 2B), indicating activation of the NLRP3 inflammasome, consistent with the appearance of cholesterol crystals. Nevertheless, our results suggest that excess cholesterol deposition is associated with lysosomal membrane rupture and late-onset NLRP3 inflammasome activation.

### Unresolved excess cholesterol accumulation in spinal cord lesions

Cholesterol cannot be catabolized in most mammalian cells; therefore, excess cholesterol must be exported to extracellular high-density lipoprotein (HDL) particles. This process is mediated by ATP-binding cassette (ABC) transporters [16]. Since liver X receptor (LXR), a nuclear receptor, regulates the expression of genes involved in cholesterol efflux, including *Abca1*, *Abcg1*, and *Apoe*, in atherosclerosis and aged mice [28–31], we examined whether the LXR agonist, GW3965, promotes cholesterol efflux from spinal cord lesions. However, oral administration of GW3965 for 4 weeks did not reduce the amounts of cholesterol crystals or oil red O (ORO)-stained lipid droplets, where esterified cholesterol was stored (Fig. 2C to F and Fig. S2A). In addition, treatment with GW3965 failed to improve locomotor recovery (Fig. S2B). As the transcription of *Abca1* was up-regulated from 7 dpi (Fig. 1B), we quantified the ABCA1 protein by western blotting analysis. Our results showed that ABCA1 protein levels substantially increased at 2 wpi and were maintained for up to 6 weeks (Fig. 2B). These data suggest that macrophages do not lack the capacity for cholesterol export in spinal cord lesions but have defects in other elements for cholesterol transportation, such as misregulated cholesterol carriers.

Apolipoprotein E (APOE) is regarded as a major CNS carrier of cholesterol for delivery from astrocytes to neurons. After spinal cord injury, the expression of *Apoe* was up-regulated (Fig. 1B). Therefore, we examined the role of APOE in cholesterol clearance in spinal cord lesions. Unexpectedly, no substantial differences were noted in the deposition of cholesterol crystals and lipid droplets between APOE knockout (KO) and wild-type (WT) mice after spinal cord injury (Fig. 2C to F). In addition, locomotion recovery was not affected in APOE KO mice (Fig. S2B). Our results suggest that APOE is not efficient or timely in removing excess cholesterol from spinal cord lesions.

### Excess cholesterol accumulation is resolved by RCT in the peripheral nervous system (PNS)

**Fig. 3.**
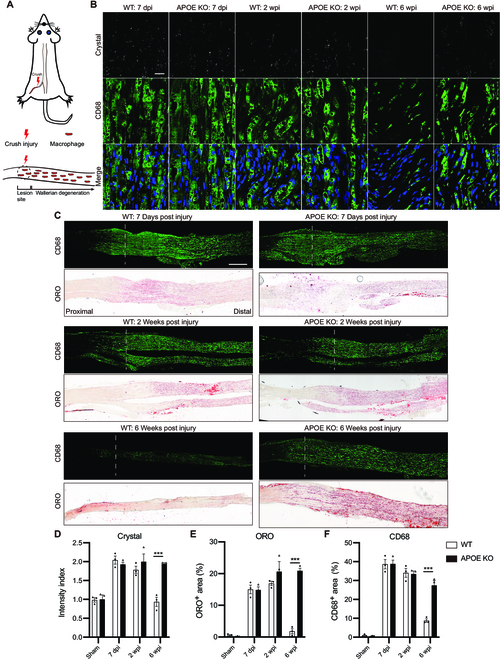
Excess cholesterol accumulation is resolved by RCT in injured sciatic nerves. (A) Schematic of sciatic nerve injury. (B) Representative images of sciatic nerves distal to the crush site at different time points after injury, showing crystal (white), CD68 (green), and DAPI (blue). (C) Representative images of sciatic nerves stained with CD68 (green) and ORO (red) at different time points after injury. The dashed line indicates the injury site. (D to F) Quantification of the crystal intensity (normalized to the proximal region), ORO-positive area (%) and CD68-positive area (%) in sciatic nerves distal to the injury site (*n* = 3 mice), Ordinary 2-way ANOVA with Tukey’s multiple comparisons test. All data are shown as mean ± SEM. ***P < 0.001. Scale bars: 20 μm (B) and 500 μm (C).

**Fig. 4.**
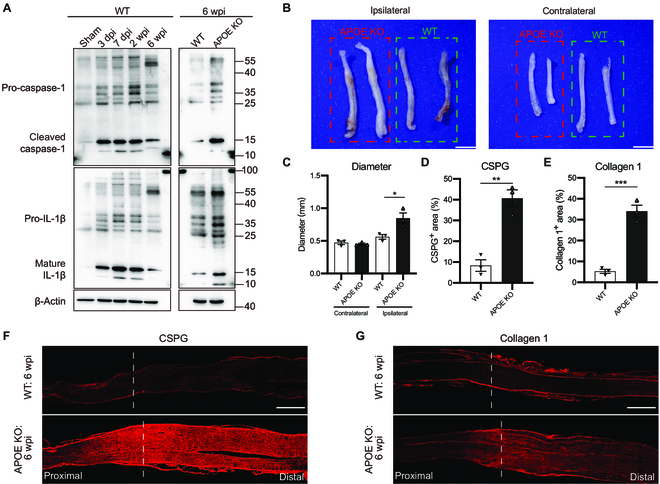
Efficient cholesterol clearance prevents NLRP3 inflammasome activation and fibrosis. (A) Immunoblots of sciatic nerves distal to the injury site for caspase-1, IL-1β, and β-actin (loading control). (B) Images of ipsilateral and contralateral sciatic nerve of APOE KO mice and WT mice at 6 wpi. (C to E) Quantification of the diameter, CSPG, and collagen 1-positive area (%) in sciatic nerves distal to the injury site (*n* = 3 mice). (F and G) Representative images of sciatic nerves stained with CSPG and collagen 1 at 6 wpi. The dashed line indicates the injury site. (C) Ordinary 1-way ANOVA with Tukey’s multiple comparisons test. (D and E) Two-tailed Student *t* test. Data are shown as mean ± SEM. *P < 0.05, **P < 0.01, ***P < 0.001. Scale bars: 500 μm (F and G) and 2 mm (B).

Peripheral nerve injury leads to Wallerian degeneration (Fig. 3A), which produces massive myelin debris distal to the site of injury. However, in contrast to lesions in the CNS, the vast majority of myelin debris in peripheral nerve injury is rapidly removed by both Schwann cells and macrophages [32,33]. We wondered whether cholesterol accumulation plays a role in the healing outcomes of CNS and PNS injuries. We first investigated whether excess cholesterol accumulation occurred after peripheral nerve injury. At 7 dpi and 2 wpi, we observed accumulation of cholesterol crystals and lipid droplets in the sciatic nerve distal to the crush site (Fig. 3B to E). Subsequently, cholesterol crystals and lipid droplets were eliminated at 6 wpi (Fig. 3B to E). Corresponding to cholesterol crystal deposition, active caspase-1 and mature IL-1β initially increased after injury and then decreased at 6 wpi (Fig. 4A). These results suggest that excess cholesterol does accumulate in the injured peripheral nerves and is subsequently removed.

As peripheral nerves lack blood–tissue barriers, excess cholesterol can be transported to the liver via plasma HDL by RCT. Given that APOE is necessary for RCT of macrophages in vivo [34,35], we analyzed the cholesterol clearance in injured nerves of APOE KO mice. In APOE KO mice, excess cholesterol was deposited in injured nerves distal to the injury site and maintained for at least 6 wpi (Fig. 3B to E), suggesting limited clearance of myelin-derived cholesterol, which was not detected in the sham group of WT and APOE KO mice (Fig. 3D and E and Fig. S3). Furthermore, compared with WT mice, both cleaved caspase-1 and mature IL-1β increased at 6 wpi in the injured nerves of APOE KO mice (Fig. 4A). These results suggest that RCT is essential for efficient cholesterol clearance in injured peripheral nerves.

### Efficient cholesterol clearance prevents macrophage accumulation and fibrosis in injured peripheral nerves

Next, we investigated the consequences of excess cholesterol accumulation following nerve repair in APOE KO mice. Consistent with excess cholesterol accumulation, injured nerves distal to the crush site were enlarged at 6 wpi in APOE KO mice but not in WT mice (Fig. 4B and C). Meanwhile, a large amount of CD68-positive macrophages accumulated in the injured nerves of APOE KO mice, whereas much less accumulated in WT mice (Fig. 3C and F). Furthermore, fibrosis was increased, as shown by chondroitin sulfate proteoglycan (CSPG) and collagen 1 staining, though not fibronectin, in the injured nerves of APOE KO mice (Fig. 4D to G and Fig. S4C and D). In addition, the density of nerve fibers stained with neurofilament 200 was significantly reduced, similar to a previous study [36], and a higher percentage of non-neural tissue, primarily macrophages, was present in the injured nerves of APOE KO mice (Fig. S4A and B). Although APOE is not essential for nerve regeneration and remyelination in APOE KO mice [36–38], our results suggest a critical role for APOE in efficient cholesterol clearance through RCT, which prevents macrophage accumulation and fibrosis in injured peripheral nerves.

### Serum is efficient for the clearance of myelin-derived cholesterol in vitro

For a better understanding of the different consequences of cholesterol accumulation in the CNS and PNS, we performed an in vitro experiment to study the role of serum in the clearance of myelin-derived cholesterol.

**Fig. 5.**
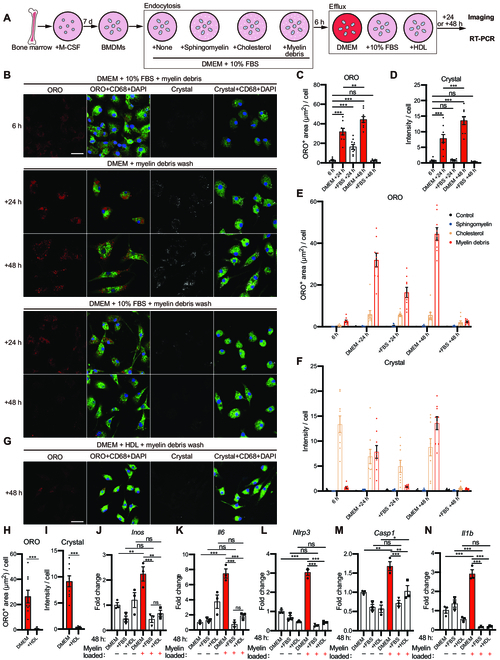
Serum is efficient to remove myelin-derived cholesterol from macrophages in vitro. (A) Schematic diagram of in vitro cholesterol efflux study. (B) Representative images of myelin-overloaded BMDMs showing ORO (red), CD68 (green), and crystal (white) in different media at different time points. (C and D) Quantification of the ORO-positive area and crystal intensity per cell after myelin treatment at different time points. (E and F) Quantification of the ORO-positive area and crystal intensity per cell after indicated treatments at different time points. The data of myelin treatment are also shown in (C) and (D) (*n* = 9 from 3 cultures). (G) Representative images of myelin-overloaded BMDMs showing nearly no deposition of ORO-positive lipid droplets (red) and crystals (white) in the CD68-positive macrophages (green) after culturing in DMEM supplemented with HDL for 48 h. (H and I) Quantification of the ORO-positive area and crystal intensity per cell in (D) (*n* = 9 from 3 cultures). (J to N) Real-time PCR analysis of the expression of *Inos*, *IL6*, *Nlrp3*, *Casp1*, and *Il1b* after myelin treatment in the indicated medium at 48 h (*n* = 3 cultures). (C, D, and J to N) Ordinary 1-way ANOVA with Tukey’s multiple comparisons test. (H and I) Two-tailed Student *t* test. All data are shown as mean ± SEM. *P < 0.05, **P < 0.01, ***P < 0.001. Scale bar: 20 μm (B and G). M-CSF, macrophage colony-stimulating factor; RT-PCR, real-time PCR.

The serum contains HDL and other cholesterol-binding agents, which stimulate cholesterol secretion in cultured macrophages [39,40]. To study the process of cholesterol efflux in response to myelin overloading, we initially incubated BMDMs with myelin debris for sufficient endocytosis of cholesterol (Fig. 5A). Subsequently, BMDMs were enlarged, and a few lipid droplets and cholesterol crystals appeared (Fig. 5B to D), suggesting that myelin debris was internalized by BMDMs and that cholesterol was already partially released from degrading myelin debris. The myelin-loaded cells were then cultured in Dulbecco’s modified Eagle’s medium (DMEM) supplemented with or without 10% fetal bovine serum (FBS), after washing off the free myelin debris (Fig. 5A). In the absence of FBS, a large number of lipid droplets and cholesterol crystals appeared in BMDMs after 24 h and persisted for up to 48 h. However, in the presence of FBS, despite the presence of lipid droplets in BMDMs at 24 h of culture, neither lipid droplets nor cholesterol crystals were detectable after 48 h (Fig. 5B to D). Besides, as a positive control, cholesterol treatment led to cholesterol deposition in BMDMs, which was subsequently eliminated by FBS (Fig. 5E and F and Fig. S5C). As a negative control, sphingomyelin, a membrane lipid, did not contribute to the formation of lipid droplets or cholesterol crystals (Fig. 5E and F and Fig. S5B). Furthermore, we cultured myelin-overloaded BMDMs in DMEM supplemented with HDL and observed that myelin-derived cholesterol was efficiently removed after 48 h (Fig. 5G to I). Taken together, our results suggest that myelin-derived cholesterol cannot be efficiently removed from macrophages unless an effective acceptor of cholesterol is present in the culture medium.

Previous studies indicated that myelin debris is involved in macrophage polarization [20,41]. Next, we analyzed macrophage polarization and the related inflammation in response to excess cholesterol accumulation. As described above, myelin-overloaded BMDMs were cultured in different media without any stimulators for 48 h (Fig. 5A). In response to excess cholesterol accumulation, BMDMs up-regulated the expression of both *Il6* and *Inos*, which are related to proinflammatory factors of M1 macrophages, and the up-regulation was reversible with the removal of accumulated cholesterol by FBS or HDL (Fig. 5J and K). However, excess cholesterol accumulation did not influence the expression levels of *Tnf*, an M1 macrophage marker, and *Arg1*, *Tgfb*, *Igf1*, and *Il10*, M2 macrophage markers (Fig. S5D to H). Furthermore, we found that the expression of *Nlrp3*, *Casp1*, and *Il1b* was up-regulated in response to excess cholesterol accumulation, but none of them were up-regulated in the presence of FBS or HDL (Fig. 5L and M). This result suggests that the increased expression of NLRP3 inflammasome components induced by excess cholesterol accumulation was rescued when cholesterol was removed. Moreover, excess cholesterol accumulation is suggested to induce specific proinflammatory responses in macrophages but not in M1/M2 activation [23].

### Myelin-derived cholesterol leads to persistent macrophage activation and scar formation in spinal cord lesions

**Fig. 6.**
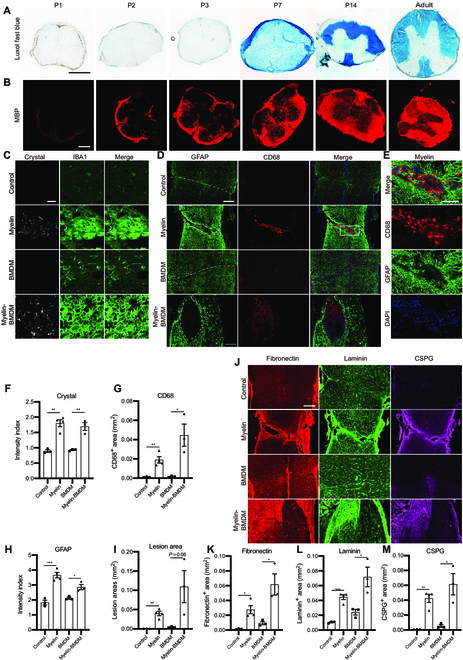
Excess accumulation of myelin-derived cholesterol leads to scar formation. (A and B) Representative images of postnatal and adult spinal cords stained with LFB (blue) and MBP (red), respectively. (C to E) Representative images of spinal cord sections at 2 weeks after transplantation of vehicle, myelin, BMDMs, or Myelin-BMDMs into the P2 injury site. (C) Representative images showing crystal (white) and IBA1 (green). (D) Representative images stained with GFAP (green), CD68 (red), and DAPI (blue). The dashed line indicates the lesion site. (E) Higher-magnification images from the boxed area in (D) showing the CD68-positive and GFAP-negative non-neural lesion core (red) and GFAP-positive glial scar (green). (F to I) Quantification of the crystal intensity (normalized to the intact region) and CD68-positive area in the lesion, and the GFAP intensity (normalized to the intact region) at the lesion border at 2 wpi (*n* = 3, 4, 3, and 3 mice for Control, Myelin, BMDMs, and Myelin-BMDMs, respectively). (J) Adjacent sections of spinal cord stained with fibronectin (red), laminin (green), and CSPG (magenta) at 2 weeks after transplantation of vehicle, myelin, BMDMs, or Myelin-BMDMs into the P2 injury site. (K to M) Quantification of the fibronectin, laminin, and CSPG-positive area in the lesion at 2 wpi (*n* = 3, 4, 3, and 3 mice for Control, Myelin, BMDMs, and Myelin-BMDMs, respectively). (F to I and K to M) Two-tailed Student *t* test for control vs. myelin and BMDMs vs. Myelin-BMDMs. All data are shown as mean ± SEM. *P < 0.05, **P < 0.01, ***P < 0.001, Scale bars: 200 μm (A, B, D, and J), 20 μm (C), and 100 μm (E).

Spinal cord injuries produce myelin and other biological membrane debris. Considering that biological membranes contain abundant lipids, including cholesterol, we asked whether myelin-derived cholesterol was necessary for cholesterol accumulation following spinal cord injury. Using luxol fast blue (LFB) to stain myelin lipids, we observed that myelin lipids were not present in the postnatal day 1 to 3 (P1 to P3) mouse spinal cord but were abundant in the spinal cord after P7, as myelination proceeded (Fig. 6A and Fig. S6A). We detected the expression of myelin basic protein (MBP), a myelin-related protein, in the mouse spinal cord starting at P2 (Fig. 6B and Fig. S6B).

Next, we induced P2 injuries to further study the roles of myelin-derived lipids [12]. We found that at 2 weeks post P2 crush injury, spinal cord lesions were repaired, without deposition of cholesterol crystals (Fig. 6C). We then immediately injected heat-inactivated (95 °C, 15 min) myelin debris into the lesion site after a P2 crush injury. As expected, we detected cholesterol crystals deposited in macrophages at the lesion core 2 weeks after myelin debris injection (Fig. 6C and F). Meanwhile, myelin injection led to a substantial accumulation of IBA1-MAC2-CD68-positive macrophages (activated macrophages) at the lesion site, and a scar was formed (Fig. 6D to I and Fig. S6C to E). Simultaneously, we observed fibrosis stained with fibronectin, CSPG, and laminin after myelin debris injection (Fig. 6J to M). These results suggest that cholesterol derived from myelin debris contributes to excess cholesterol accumulation and scar formation in neonatal lesions, similar to adult lesions.

Immune cell infiltration has been reported to drive CNS fibrosis by proliferative fibroblasts [9]. To determine the role of cholesterol-overloaded macrophages in scar formation, we initially overloaded BMDMs with cholesterol by incubating them with myelin debris and then transplanted the myelin-overloaded BMDMs into the P2 injury site. At 2 weeks post transplantation, cholesterol-overloaded BMDMs closely clustered in the lesion, surrounded by astrocytes (Fig. 6C, D, F to I, and Fig. S6C to E). Similarly, fibrosis was detected after transplantation (Fig. 6J to M). As a control, transplantation of untreated BMDMs did not result in cholesterol crystal deposition, active macrophage accumulation, or fibrosis (Fig. 6C, D, and F to M and Fig. S6C to E). These results suggest that excess cholesterol accumulation contributes to persistent macrophage activation, which impairs complete healing and promotes scar formation.

Taken together, our results suggest that myelin-derived cholesterol not only results in excess cholesterol deposition but also contributes to impaired scar-free healing with macrophage activation and fibrosis in neonatal spinal cord lesions.

### Myelin phagocytosis suppresses macrophage apoptosis

**Fig. 7.**
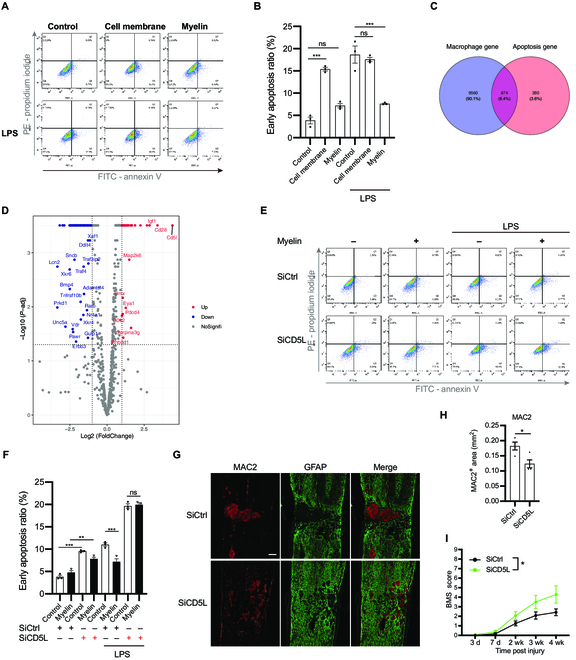
Myelin-loaded macrophages increased apoptosis resistance. (A) FACS plots showing the macrophage apoptosis ratio after indicated treatments using annexin V/propidium iodide apoptosis assay. (B) Quantification of apoptosis ratio in (A). (C) Venn plot of macrophage genes in injured spinal cords and apoptosis genes selected from GO. (D) Volcano plot of apoptosis genes expressed in macrophages. Up indicates that gene expression is elevated at 7 dpi compared to its expression at 3 dpi. (E) FACS plots showing macrophage apoptosis ratio after indicated treatments using annexin V/propidium iodide apoptosis assay. (F) Quantification of apoptosis ratio in (E). (G) Representative confocal images showing MAC2 (red) and FGAP (green) taken at 4 wpi. Scale bar: 200 μm. (H) Quantification of the MAC2-positive area in (G) (*n* = 4 mice). (I) Evaluation of locomotor recovery using the BMS scores (*n* = 7 mice). (B and F) Ordinary 1-way ANOVA with Tukey’s multiple comparisons test. (H) Two-tailed Student *t* test. (I) Repeated measures 2-way ANOVA with the Greenhouse–Geisser correction. All data are shown as mean ± SEM. *P < 0.05, **P < 0.01, ***P < 0.001. PI, propidium iodide; FITC, fluorescein isothiocyanate.

Macrophages are timely eliminated after non-neuronal injuries, which are pivotal for the resolution of inflammation [42]. Apoptosis of inflammatory macrophages has been reported to promote macrophage clearance during resolution [43]. To determine whether myelin phagocytosis restricted macrophage apoptosis, we incubated BMDMs with myelin debris that was rich in cholesterol and cell membrane debris isolated from P2 spinal cords that was less in cholesterol. Apoptosis assays using fluorescence-activated cell sorting (FACS) indicated that myelin-loaded BMDMs were less prone to apoptosis than cell membrane-loaded BMDMs, spontaneously or stimulated with lipopolysaccharide (LPS) (Fig. 7A and B), suggesting that myelin internalization suppresses macrophage apoptosis.

Using a macrophage-specific transcriptional profile directly from the adult injured spinal cords at 3 and 7 dpi [23], we analyzed macrophage genes, all of which were annotated as apoptotic processes in GO (Fig. 7C). The volcano plot showed that macrophages obviously up-regulated *Cd5l* expression at 7 dpi, when they had accumulated excessive cholesterol, compared with that at 3 dpi. CD5L, which is mainly expressed by macrophages, is responsible for preventing macrophage apoptosis, also known as apoptosis inhibitor of macrophages [44]. Moreover, prior studies have revealed that CD5L is regulated by cholesterol metabolism and promotes atherosclerosis [45,46]. Therefore, we verified that CD5L expression was elevated in macrophages in spinal cord lesions at 7 dpi (Fig. S7A and E). Myelin internalization also increased CD5L expression in BMDMs, whereas incubation with cell membrane debris decreased CD5L expression (Fig. S7B and F). Transfection of BMDMs with the small interfering RNA (siRNA) targeting CD5L (SiCD5L) expectedly promoted apoptosis. Furthermore, treatment of BMDMs with SiCD5L reversed the suppression of apoptosis induced by myelin phagocytosis after LPS stimulation (Fig. 7E and F and Fig. S7C and G). The delivery of SiCD5L into the injured spinal cord starting at 7 dpi showed less macrophage deposition and better locomotor recovery than the delivery of SiCtrl (Fig. 7G to I and Fig. S7D and H). These data suggest that CD5L is involved in resistance to apoptosis in myelin-loaded macrophages and is a potential target for intervention in spinal cord injury.

## Discussion

In CNS lesions, scar formation impairs spontaneous wound healing. Here, we revealed an intrinsic mechanism that contributes to scar formation. Unresolved excessive myelin-derived cholesterol accumulates in the spinal cord lesions, inducing persistent macrophage activation and scar formation. By contrast, efficient cholesterol clearance by RCT prevents macrophage accumulation and fibrosis in injured peripheral nerves.

Pharmacological stimulation has been reported to rescue cholesterol export deficiency of phagocytes both in vitro and in vivo [18,47,48]. However, the administration of GW3965, an LXR agonist, failed to rescue excess cholesterol accumulation in spinal cord lesions in young adult mice. Our data suggest that macrophages in spinal cord lesions do not lack cholesterol export capacity but lack other elements for cholesterol transportation.

As an in vivo example, in injured peripheral nerves, excess cholesterol is efficiently removed by RCT, while RCT is absent in the CNS. Furthermore, our in vitro experiments demonstrated that myelin-derived cholesterol could not be efficiently removed from macrophages unless an effective cholesterol acceptor was present. Beyond the perspective of this study, adiponectin was reported to reduce myelin lipid accumulation after spinal cord injury [49]. In addition, deficiency of cholesterol import-related receptors partially reduces the formation of foamy macrophages [23,50]. Thus, developing an efficient strategy for excess cholesterol removal or recycling may rescue impaired healing of CNS lesions.

Although the role of APOE in CNS diseases has been widely explored, the potential influence of APOE on peripheral nerve injury remains incompletely understood [36–38,51–56]. Here, we unveiled the role of APOE in cholesterol clearance following peripheral nerve injury. APOE plays a role in RCT [34,35] by efficiently removing myelin-derived cholesterol produced by Wallerian degeneration. Remarkably, excess cholesterol accumulation was related to macrophage accumulation and fibrosis in the peripheral nerve of APOE KO mice.

Recent research has identified *Apoe* as a hub gene after spinal cord injury [57], suggesting the importance of cholesterol homeostasis in spinal cord lesions. Our current study focuses on the phase of scar formation. Cholesterol clearance is expected to be timely and effective to resolve the healing process, but our results suggest that the role of APOE in cholesterol clearance in our model is not timely and efficient. This discrepancy may be due to the much larger lesion (more than 10-fold) in our study than the demyelinating lesion induced by lysolecithin injection, which implied a requirement for APOE [18]. APOE possibly participated in cholesterol clearance in spinal cord lesions, but the difference was not sufficient to be detected, considering that the lesion size was large in the injury model we used. The effect of APOE on cholesterol clearance can possibly be detected in the long-term phase after spinal cord injury.

The pivotal role of lipid homeostasis in spinal cord lesions has been noted and revealed in recent studies [20,23,57–59]. Our study focused on cholesterol homeostasis and found that the formation of cholesterol crystals at CNS lesion sites induces inflammasome activation. Other cholesterol-induced toxicities may also participate in the pathological process of CNS injury, such as cholesterol-induced inflammation, endoplasmic reticulum stress, oxidative stress, and mitochondrial dysfunction [15,60,61]. In addition to the macrophages we showed in this study, microglia, astrocytes, and even microvascular endothelial cells may also be challenged by the accumulation of excess cholesterol derived from myelin [57,62]. Besides, other lipids such as triglycerides may be involved in the pathological process after spinal cord injury. Nevertheless, this study provides a foundation for further exploration of the consequences of excess cholesterol accumulation in the CNS.

The neonatal (P2) spinal cord lacks myelin-derived lipids and can be completely repaired without scar formation after injury [12]. This provided us with a model to test the role of myelin-derived lipids. We showed that similar to adult spinal lesions [20], cholesterol-overloaded macrophages persist in neonatal lesions after transplantation of myelin or myelin-overloaded BMDMs, leading to scar formation instead of healing. Consistently, reduced cholesterol clearance in APOE KO mice inhibited peripheral nerve healing. Notably, in P2 neonatal injury, transient activation of BMDMs and microglia is induced at 3 dpi but is eliminated by 2 wpi [12]. Moreover, myelin-overloaded macrophages are less prone to apoptosis than cell membrane-overloaded macrophages, which is mediated by CD5L expression. Although the underlying mechanism of macrophage elimination in cholesterol-less wounds remains to be elucidated, our results suggest that excess cholesterol accumulation contributes to scar formation mediated by persistent macrophage activation. Further studies are needed to examine whether reducing excess cholesterol in macrophages can reduce scarring in spinal cord lesions. In addition, whether other variables, such as age and immune response, contribute to cholesterol homeostasis after neonatal spinal cord injury is unknown.

Our study highlights that excess myelin-derived cholesterol accumulation contributes to persistent activation of macrophages and scar formation after spinal cord injury. Since efficient cholesterol clearance by RCT prevents macrophage accumulation and fibrosis in injured peripheral nerves, this study suggests that promoting cholesterol clearance and reestablishing homeostasis of cholesterol-overloaded macrophages are potential strategies to facilitate scarless healing in the CNS.

## Materials and Methods

### Animals

All animal experiments were performed in compliance with protocols approved by the Institutional Animal Care and Use Committees of Tongji University. All mice were housed under temperature-controlled conditions on a 12-h light-dark cycle, with ad libitum feeding. C57BL/6 and APOE KO mice purchased from Shanghai Jiesijie Laboratory were used in this study. APOE KO mice were maintained on C57BL/6 genetic background. Genotype identification was performed using the following primers: common forward GCC TAG CCG AGG GAG AGC CG; WT reverse TGT GAC TTG GGA GCT CTG CAG C, and mutant reverse GCC GCC CCG ACT GCA TCT.

### Spinal cord injury

A spinal cord contusion injury was conducted in 8- to 12-week-old female mice at thoracic level 10 (T9). Briefly, mice were anesthetized with isoflurane (2% to 3%), and the thoracic vertebrae were exposed, followed by a T9 laminectomy. The mice were then stabilized, and a moderate contusion was performed using an NYU/MASCIS (New York University, Multicenter Animal Spinal Cord Injury Study) impactor with a height of 12.5 mm. The muscles and the skin were then sutured. The bladders were manually expressed 2 times per day.

Neonatal crush injury was induced in P2 mice at T10. The spinal cord was exposed entirely and was then crushed for 3 s using a Dumont No. 5 forceps. The muscles and the skin were then sutured. Neonatal mice were warmed up and rubbed with the urine from the mom at the skin wound. Phosphate buffer saline (PBS, 0.5 μl), heat-inactivated myelin debris (0.5 μl, protein concentration: 4.23 μg/μl), and BMDMs or myelin-overloaded BMDMs (1 × 10^5^) were immediately injected into the lesion site after a crush injury.

### Sciatic nerve crush injury

Sciatic nerve injuries were conducted in 8- to 12-week-old female mice. Mice were anesthetized with isoflurane (2% to 3%). After the hind limb area was shaved, an incision was made at the left thigh, and sciatic nerves were gently exposed and crushed for 30 s using a Dumont No. 5 forceps (at 1.5 mm from the tip). After crushing, the crushed site was translucent. The wound was then closed using sutures.

### Drug administration

For LXR agonist administration, mice were fed with powdered standard rodent chow supplemented with GW3965 (20 mg/kg), according to a previous study [18]. Administration was immediately started after the injury and continued for 4 weeks. For siRNA delivery, SiCD5L or SiCtrl (1 μl, 2.5 nmol) was injected into the center of the injury site using a microsyringe starting at 7 dpi and repeated every 3 d until 4 times.

### Behavioral evaluations

We evaluated locomotor recovery when mice were walking in an open field using the Basso mouse scale (BMS) scores at 3 d after spinal cord injury and weekly thereafter [63]. The mice were evaluated without any stimulation for 5 min after adaptation to the environment for 2 min. The evaluation was performed by 2 experimenters in a blinder way.

### Immunohistochemistry and imaging

After anesthesia with an overdose of sodium pentobarbital, the mice were transcardially perfused with PBS followed by 4% paraformaldehyde. Spinal cords and sciatic nerves were dissected and fixated further by immersion in 4% paraformaldehyde at 4 °C overnight and subsequently dehydrated in 30% buffered sucrose for 24 h. Equilibrated tissues were embedded in optimal cutting temperature for longitudinal sectioning and stored at −80 °C until processing. Thick frozen sections (20 μm) were prepared using a Leica CM3050S cryostat microtome. Longitudinal sections of the spinal cord were numbered in order from the ventral to the dorsal side, and sections of the same/adjacent number from different mice were used for each staining.

For immunohistochemical analysis, the sections were permeabilized and blocked with blocking buffer (0.1% Triton, 0.05% Tween 20, and 10% normal donkey/goat serum in PBS) for 1 h at room temperature before primary antibody incubation. The following primary antibodies were used: rat anti-CD68 (1:1,000, ab53444; Abcam), rabbit anti-IBA1 (1:1,000, 019-19741; Wako), rabbit anti-glial fibrillary acidic protein (GFAP) (1:1,000, Z0334; Dako), goat anti-GFAP (1:1,000, ab53554; Abcam), rat anti-MAC2 (1:500, 125401; BioLegend), rabbit anti-LAMP1 (1:200, bs-1970R, Bioss), rat anti-CD107a (1:1,000, CL488-65050, Proteintech), rabbit anti-fibronectin (1:200, F3648; Sigma-Aldrich), rabbit anti-laminin (1:200, L9393; Sigma-Aldrich), mouse anti-chondroitin sulfate (1:200, c8035; Sigma-Aldrich), rat anti-MBP (1:200, MAB386; Sigma-Aldrich), mouse anti-CD5L (1:200, sc-390486; Santa Cruz Biotechnology), rabbit anti-collagen1 (1:200, 72026; Cell Signaling Technology). The secondary antibodies were Alexa Fluor 488- and Alexa Fluor 555-conjugated donkey anti-goat/rabbit antibodies (1:500; Invitrogen) and Alexa Fluor 488 and Alexa Fluor 594-conjugated goat anti-rat/rabbit/mouse antibodies (1:500; Invitrogen). Sections were counterstained with 4′,6-diamidino-2-phenylindole (DAPI) and mounted using Fluoromount-G antifade mounting medium (Southern Biotechnology). Tissue sections were imaged using a confocal laser scanning microscope (LSM 880; Zeiss), and the sections of each experimental group were photographed using consistent exposure settings. For MBP staining, the sections were pretreated with 95% ethanol for 10 min.

### Crystal imaging

Confocal reflection microscopy, which detects backscattered light to create high-resolution images, was used to visualize cholesterol crystals as described previously [17]. For confocal reflection imaging, frozen sections were scanned with a 405-nm laser, the pinhole size was set to 0.3 AU, and the reflected light was collected within 400 to 410 nm. Confocal reflection and fluorescence microscopy (LSM 880; Zeiss) were combined to scan the multiply labeled tissues.

### Cholesterol crystal preparation

Cholesterol crystals were prepared by dripping distilled water (0.5 ml) into a cholesterol solution in ethanol (1 ml, 2 mg/ml) and sonicating for 15 min. Then, the suspension or the mixed suspension of cholesterol crystal suspension with Fluoromount-G mounting medium (Southern Biotechnology) (2:1) was coverslipped for confocal reflectance imaging.

### Transmission electron microscopy

At 2 wpi, the spinal cord was isolated after perfusion with 4% paraformaldehyde and fixed in 2.5% glutaraldehyde. The 1-mm tissue of the lesion center was then dissected, postfixed in 1% osmium tetroxide, dehydrated through a series of graded alcohols, cleared in acetone, and embedded in Epon. Ultrathin sections (60 nm) were prepared using an ultramicrotome, collected on copper grids, and stained with uranyl acetate and lead citrate. After drying, the grids were viewed using a 120-kV transmission electron microscope (Hitachi HT 7800).

### ORO staining

To visualize neutral lipids, primarily triacylglycerol and cholesterol esters, frozen sections were rinsed with 60% isopropanol for 30 s and incubated with freshly prepared ORO working solution (G1261; Solarbio) for 15 min. Next, the slides were differentiated in 60% isopropanol, rinsed with distilled water, and mounted for further imaging.

### LFB staining

Frozen sections were incubated in LFB solution (G3245; Solarbio) for 24 h at room temperature. Sections were then rinsed in water, differentiated in lithium carbonate solution for 15 s, and differentiated further in 70% alcohol reagent for 30 s. Subsequently, the sections were rinsed with water and mounted.

### RNA isolation and real-time PCR

For expression analysis, a 3-mm spinal cord of the lesion center was dissected and homogenized in RNAiso Plus (9108; Takara) using a cryogenic grinder (Shanghai Jingxin JXFSTPRP-CL). Total RNA was isolated according to the manufacturer’s manual, and the RNA concentration and quality were measured using a NanoDrop spectrophotometer (Thermo Fisher Scientific). After cDNA synthesis with a PrimeScript RT Master Mix (RR036A, Takara), real-time PCR was performed using TB Green Premix Ex Taq II (RR820A; Takara) on a QuantStudio 7 Flex real-time PCR system (Thermo Fisher Scientific). All the primers used are listed in Table S1. Using the ΔΔ−Ct method, the expression levels of target genes were calculated (β-actin as a housekeeping gene, sham group as reference samples).

### Western blot

Spinal cords, 3 mm from the lesion center, and sciatic nerves, 5 mm distal to the crush site, were extracted and homogenized in radioimmunoprecipitation assay lysis buffer (P0013B; Beyotime) supplemented with protease and phosphatase inhibitor cocktail and 5 mM EDTA (P1051; Beyotime). The supernatants of tissue lysates were collected after centrifugation (12,000 g for 20 min at 4 °C), and protein concentrations were measured using a protein BCA Protein Assay Kit (KGP903; Keygen). Total protein (20 μg) was separated by sodium dodecyl sulfate-polyacrylamide gel electrophoresis and transferred to polyvinylidene difluoride membranes. The membranes were blocked with 5% milk at room temperature for 1 h and then incubated with primary antibody overnight at 4 °C. The following primary antibodies were used: rabbit anti-ABCA1 (1:1,000, bs-23418R; Bioss), rabbit anti-caspase-1 (1:1,000, 22915-1-AP; Proteintech), rabbit anti-IL-1β (1:1,000, 16806-1-AP; Proteintech), rabbit anti-CTSD (1:1,000, 21327-1-AP; Proteintech), and rabbit anti-β-actin (1:1,000, 8457; Cell Signaling Technology). After washing with TBST, the membranes were incubated with horseradish peroxidase-conjugated goat anti-rabbit IgG (1:3,000, ab6721; Abcam) at room temperature for 1 h. Membranes were visualized using chemiluminescence (WBKLS0500; Millipore).

### Myelin and cell membrane isolation

Myelin was extracted from adult C57BL/6 mouse brains by sucrose-gradient centrifugation as previously described [64], with some modifications [65]. The cell membrane was extracted from P2 neonatal spinal cords using 0.32 M sucrose as previously described [66]. At the last step, the purified myelin or cell membrane fraction was resuspended in PBS and stored at −80 °C. The total protein concentration was measured using a protein BCA protein assay kit (KGP903; Keygen).

### BMDM isolation and culture

To prepare BMDMs, 8-week-old mice were sacrificed by cervical dislocation and soaked in 75% ethanol. Then, the femur and tibia were exposed, dissected, and flushed with DMEM using a 2-ml syringe. The bone marrow cells were then filtered through a 100-μm cell strainer and cultured in basal medium (DMEM supplemented with 1% GlutaMAX and 1% penicillin–streptomycin) supplemented with 20 ng/ml macrophage colony-stimulating factor and 10% FBS for at least 7 d. For endocytosis, mature BMDMs were cultured in a basal medium supplemented with 10% FBS and incubated with myelin debris (100 μg myelin protein per millimeter), cholesterol (100 μg/ml), and sphingomyelin (100 μg/ml) for 6 h. The media were sonicated for 15 min before addition. For the study of cholesterol efflux, cells were then cultured in basal medium, basal medium + 10% FBS and basal medium + HDL (0.1 mg/ml) for an additional 24 h or 48 h.

### Apoptosis analyses

BMDMs were incubated with myelin debris (100 μg protein per ml) or cell membrane debris (100 μg protein per ml) for 2 h, cultured for 24 h to digest debris in serum-free medium, and then stimulated with LPS (100 ng/ml) for an additional 24 h. Cells were then collected for apoptosis analysis using the Annexin V-fluorescein isothiocyanate apoptosis detection kit with propidium iodide (Beyotime; C1062L) and a BD LSRFortessa Cell Analyzer. For knockdown of CD5L, BMDMs were transfected with siRNA against CD5L or control siRNA (100 pmol) using Lipofectamine 3000 (Invitrogen) before the apoptosis tests. siCD5L was purchased from Generay Biotechnology and the sequence (5′–3′) was listed as follows: GATCGTGTTTTTCAGAGTCTCCA.

### Bioinformatic analyses

GSE84737, available at Gene Expression Omnibus, describes the macrophage-specific transcriptional profile directly from the injured adult spinal cord at 3 and 7dpi using the RiboTag method, and the supplementary file of differentially expressed genes (filtered) was used for analysis. All genes annotated to the apoptosis process were selected from GO. A volcano plot was constructed with R.

### Quantification

All images were measured using the Fiji software. During preparation of the longitudinal tissue sections, each section was numbered in order, and sections of similar position/level from different mice were selected as described above. One section in the center of each individual was used for staining and measurement, and the number of mice analyzed is described in the figure legends. For quantification of ORO-, CD68-, CSPG-, fibronectin-, collagen 1-, and neurofilament 200-positive area (%) in sciatic nerve sections, the average value of each section was measured with 3 fields (300-μm-wide squares), which were selected along the crush-distal axis from 500 μm distal to the crush site (Wallerian degeneration area), every 300 μm apart. For quantification of ORO-positive area and crystal intensity of BMDMs in vitro, 3 fields from each culture (3 cultures) were measured and normalized to cell numbers. For quantification of CD68, IBA1 MAC2, fibronectin, laminin, and CSPG in neonatal spinal cord lesions, the positive area was measured at the lesion site, and the GFAP intensity was measured at the border. The spinal cord lesion was defined as the GFAP-negative region. For quantification of crystal in tissues, the intensity of backscattered signals was measured in the indicated area (lesion core or lesion border of adult spinal cord lesions, Wallerian degeneration area of injured sciatic nerves), and the intensity index was calculated by normalizing to the proximal /intact region of the spinal cord or sciatic nerve [12].

### Statistical analysis

All data analyses were performed using GraphPad Prism. The number of samples is described in the figure legends, and the data distribution was assumed to be normal. For comparisons between 2 columns, a 2-tailed Student *t* test was performed. For comparisons between multiple columns, ordinary 1-way analysis of variance (ANOVA) with Tukey’s multiple comparisons test was conducted. For comparison between multiple columns of grouped data (Fig. 3), ordinary 2-way ANOVA with Tukey’s multiple comparisons test was performed. For comparison of the BMS scores, repeated measures 2-way ANOVA with the Greenhouse–Geisser correction was conducted. All values are shown as means ± SEM. *P < 0.05, **P < 0.01, ***P < 0.001.

## Data Availability

The data that support the findings of this study are available from the corresponding author upon reasonable request.

## Supplementary Materials

Supplementary Materials Fig. S1. Cholesterol crystals appear in spinal cord lesions. Fig. S2. Evaluation of interventions. Fig. S3. No deposition of cholesterol in uninjured sciatic nerves. Fig. S4. Limited cholesterol clearance reduces nerve fiber density. Fig. S5. Deposition and removal of cholesterol from macrophages in vitro. Fig. S6. Macrophages deposit in spinal cord lesions on account of myelin-derived lipids. Fig. S7. Myelin internalization promotes the expression of CD5L Table S1. List of primer sequences for real-time PCR.
