# Supplementary material for: Unresolved Excess Accumulation of Myelin-Derived Cholesterol Contributes to Scar Formation after Spinal Cord Injury
Source: Research (Wash D C). 2023 May 4;6:0135. doi: 10.34133/research.0135 (PMC10202378; doi:10.34133/research.0135)
Supplement: Supplementary Materials — Fig. S1. Cholesterol crystals appear in spinal cord lesions. Fig. S2. Evaluation of interventions. Fig. S3. No deposition of cholesterol in uninjured sciatic nerves. Fig. S4. Limited cholesterol clearance reduces nerve fiber density. Fig. S5. Deposition and removal of cholesterol from macrophages in vitro. Fig. S6. Macrophages deposit in spinal cord lesions on account of myelin-derived lipids. Fig. S7. Myelin internalization promotes the expression of CD5L Table S1. List of primer sequences for real-time PCR. [file research.0135.f1.zip › Supplementary Table 1.pdf]

Supplementary Table 1: List of primer sequences for real-time PCR.

Gene	Forward primer (5'-3')	Reverse primer (5'-3')
Hmgcr	GCTCGTCTACAGAAACTCCACG	GCTTCAGCAGTGCTTTCTCCGT
Fdft1	GGATGTGACCTCCAAACAGGAC	CAGACCCATTGAGTTGGCACAC
Cyp51	ATCCAGAAGCGCAGGCTGTCAA	CAGTCCGATGAGCATCCCTGAT
Dhcr24	CTGGAGAACCACTTCGTGGAAG	CTCCACATGCTTGAAGAACCAGG
Sqle	TGTTGCGGATGGACTCTTCTCC	GTTGACCAGAACAAGCTCCGCA
Srebf2	AGAAAGAGCGGTGGAGTCCTTG	GAACTGCTGGAGAATGGTGAGG
Cd36	GGAGCCATCTTTGAGCCTTCA	GAACCAAACTGAGGAATGGATCT
Trem2	CTACCAGTGTCAGAGTCTCCGA	CCTCGAAACTCGATGACTCCTC
Ldlr	GAATCTACTGGTCCGACCTGTC	CTGTCCAGTAGATGTTGCGGTG
Vldlr	ACGGCAGCGATGAGGTCAACTG	CAGAGCCATCAACACAGTCTCG
Apobr	GGATGTTCACAGCAACTGGAATG	GTCACACTGTGGCTCAGGAACA
Lrp1	CGAGAGCCTTTGTGCTGGATGA	CGGATGTCCTTCTCAATGAGGG
Npc1	TGAGGTCATCCCATTCCTGGTG	TCCAGCGTTTCCTCCTGAAGAC
Npc2	GCCAGTCCTACAGTGTCAACATC	TCTTACAACCGTCAGGCTCAGG
Tspo	GAGCCTACTTTGTACGTGGCGA	GCTCTTTCCAGACTATGTAGGAG
Acat1	TGAGAGCACCTCCAGAACAAGG	GGACGAATAGGATGAGGAGTGC
Nceh1	CGGTATTTCTGGAGACAGTGCTG	GGTGTGTTGAAGTCCAAAGCCTG
Cyp46a1	CTCAGGACGATGAGGTTCTGCT	TGGCGAGACAACTCCATCACTG
Ch25h	CCTAAGTCACGTCCTGATCTGC	GAGGACGAGTTCTGGTGATGCA
Cyp27a1	ATGGGATCTTCATCGCACA	CGTTTAAGGCATCCGTGTAGA
Abca1	TGTCTGAAAAAGGAGGACAGTG	TGTCACTTTCATGGTCGCTG
Abcg1	CAGACGAGAGATGGTCAAAGA	TCAAAGAACATGACAGGCGG
Apoe	CTGACAGGATGCCTAGCC	TCCCAGGGTTGGTTGCTTTG
Apoj	GATGATCCACCAGGCTCAACAG	ACACAGTGCGGTCATCTTCACC
Apod	GGTGAAGCCAAACAGAGCAACG	CAGGAGTACACGAGGGCATAGT
Pltp	GCTGCTGAACATCTCCAACGCA	GCTGTAGACCTGTTCGGATGGA
Lcat	CAGTCCTGGAAGGACCACTTCA	GAAGTCGTGGTTATGCGCTGCT
Il6	TACCACTTCACAAGTCGGAGGC	CTGCAAGTGCATCATCGTTGTTC
Inos	GAGACAGGGAAGTCTGAAGCAC	CCAGCAGTAGTTGCTCCTCTTC
Tnf	TCTTCTCATTCCTGCTTGTGG	GGTCTGGGCCATAGAACTGA
Arg1	CATTGGCTTGCGAGACGTAGAC	GCTGAAGGTCTCTTCCATCACC
Il10	CGGGAAGACAATAACTGCACCC	CGGTTAGCAGTATGTTGTCCAGC
Igf1	GTGGATGCTCTTCAGTTCGTGTG	TCCAGTCTCCTCAGATCACAGC
Tgfb1	TGATACGCCTGAGTGGCTGTCT	CACAAGAGCAGTGAGCGCTGAA
Nlrp3	TCACAACTCGCCCAAGGAGGAA	AAGAGACCACGGCAGAAGCTAG
Casp1	GGCACATTTCCAGGACTGACTG	GCAAGACGTGTACGAGTGGTTG
Il1b	GCAACTGTTCCTGAACTCAACT	ATCTTTTGGGGTCCGTCAACT
Actb	GCTTCTAGGCGGACTGTTACTGA	GCCATGCCAATGTTGTCTCTTAT
