# Supplementary material for: Unresolved Excess Accumulation of Myelin-Derived Cholesterol Contributes to Scar Formation after Spinal Cord Injury
Source: Research (Wash D C). 2023 May 4;6:0135. doi: 10.34133/research.0135 (PMC10202378; doi:10.34133/research.0135)
Supplement: Supplementary Materials — Fig. S1. Cholesterol crystals appear in spinal cord lesions. Fig. S2. Evaluation of interventions. Fig. S3. No deposition of cholesterol in uninjured sciatic nerves. Fig. S4. Limited cholesterol clearance reduces nerve fiber density. Fig. S5. Deposition and removal of cholesterol from macrophages in vitro. Fig. S6. Macrophages deposit in spinal cord lesions on account of myelin-derived lipids. Fig. S7. Myelin internalization promotes the expression of CD5L Table S1. List of primer sequences for real-time PCR. [file research.0135.f1.zip › supplementary figure legends.docx]

**Supplementary figure legends**

**Supplementary Fig.1: Cholesterol crystals appear in spinal cord lesions**.

**a,** Quantification of the percentage of MAC2-positive cells within IBA1-positive and crystal-positive cells at the lesion core and lesion border at 2 wpi (n=4 mice). **b,** Quantification of the percentage of crystals in IBA-positive and GFAP-positive cells at the lesion core and lesion border at 2 wpi (n=4 mice). **c,** Representative reflection images of cholesterol crystals in the suspension, mounting medium, and adipose tissue; The reflection signal is shown as white. **d,** Representative images of spinal cord lesions of rat at 2 wpi, showing crystals (white) in IBA1-positive phagocytes (red). Scale bar: 20 μm (**c, d**).

**Supplementary Fig.2: Evaluation of interventions. a,** Real-time PCR analysis of *Abca1*, *Abcg1* and *Apoe* after indicated treatments (n=3 mice). Ordinary one-way ANOVA with Tukey’s multiple comparisons test. Data are shown as mean ± SEM. **p < 0.01. **b,** Evaluation of locomotor recovery using the BMS scores (n=8 mice). No significant differences were observed among the experimental groups. Repeated measures two-way ANOVA with the Greenhouse–Geisser correction. Data are shown as mean ± SEM.

**Supplementary Fig.3: No deposition of cholesterol in uninjured sciatic nerves. a**, Representative images of uninjured sciatic nerves of WT and APOE KO mice, showing crystal (white), CD68 (green), and DAPI (blue). **b**, Representative images of uninjured sciatic nerves of WT and APOE KO mice stained with CD68 (green) and ORO (red). Scale bar: 20 μm (**a**), 500 μm (**b**).

**Supplementary Fig.4: Limited cholesterol clearance reduces nerve fiber density. a**, Representative images of sciatic nerves of APOE KO and WT mice stained with CD68 (green), NF200 (red), and DAPI (blue) taken at 6 wpi. Higher magnification images from boxed areas showing the density of nerve fibers (red) and CD68-positive macrophages (green). The dashed line indicates the injury site. **b**, Quantification of the NF200-positive area in sciatic nerve distal to the injury site at 6 wpi (n=3 mice). **c**, **d**, Representative images of sciatic nerves stained with fibronectin (green) and quantification of the fibronectin-positive area (%) in sciatic nerves distal to the injury site at 6 wpi (n=3 mice). **b**, **c**, Two-tailed Student’s t-test. All data are shown as mean ± SEM. ***p < 0.001, ns: not significant. Scale bar: 500 μm (**a**, **d**), 50 μm (**a**, higher magnification images).

**Supplementary Fig.5: Deposition and removal of cholesterol from macrophages *in vitro*. a-c**, Representative images of BMDMs showing ORO (red), CD68 (green), Crystal (white), and DAPI (blue) after indicated treatments at different time points. Scale bar: 20 μm. **d-h**, Real-time PCR analysis of the expression of *Arg1, Tnf*, *Igf1*, *Il10*, and *Tgfb1* in myelin-overloaded BMDMs after incubating in the indicated medium for 48 hours (n=3 cultures). Ordinary one-way ANOVA with Tukey’s multiple comparisons test. Data are shown as mean ± SEM.

**Supplementary Fig.6: Macrophages deposit in spinal cord lesions on account of myelin-derived lipids. a**, **b**, Quantification of the LFB and MBP-positive area (%) in the spinal cord sections of postnatal and adult mice, respectively (n=3 mice). **c**, Representative images of spinal cord lesions stained with IBA1 (green), MAC2 (red), and DAPI (blue) taken at 2 wpi. Scale bar: 200 μm. **d**, **e**, Quantification of the IBA1 and MAC2-positive area in spinal cord lesions at 2 wpi (n= 3, 4, 3, and 3 mice for Control, Myelin, BMDM, and Myelin-BMDM, respectively). **a**, **b**, Ordinary one-way ANOVA with Tukey’s multiple comparisons test. **d**, **e**, Two-tailed Student’s t-test for control vs. myelin and BMDMs vs. Myelin-BMDMs. All data are shown as mean ± SEM. *p < 0.05, **p < 0.01, ***p < 0.001.

**Supplementary Fig.7: Myelin internalization promotes the expression of CD5L. a,** Representative confocal images showing CD5L (green), MAC2 (red), and DAPI (blue) at different time points after adult spinal cord crush injury. **b,** Representative images of macrophages showing CD5L (green) after indicated treatments. **c,** Representative images of macrophages showing CD5L (green) after indicated treatments. **d**, Representative confocal images showing CD5L (green) and MAC2 (red) taken at 4 wpi. **e,** Quantification of the CD5L-positive area in **a** (n=3 mice). **f,** Quantification of the CD5L intensity per cell in **b** (n=3 cultures). **g,** Quantification of the CD5L intensity per cell in **c** (n=3 cultures). **h,** Quantification of the CD5L-positive area in **d** (n=4 mice). **e, h,** Two-tailed Student’s t-test. **f, g.** Ordinary one-way ANOVA with Tukey’s multiple comparisons test. All data are shown as mean ± SEM. *p < 0.05, **p < 0.01, ***p < 0.001, ns: not significant. Scale bar: 200 μm (**a, d**), 20 μm (**b, c**).
