# Supplementary figures and images for: Unresolved Excess Accumulation of Myelin-Derived Cholesterol Contributes to Scar Formation after Spinal Cord Injury
Source: Research (Wash D C). 2023 May 4;6:0135. doi: 10.34133/research.0135 (PMC10202378; doi:10.34133/research.0135)

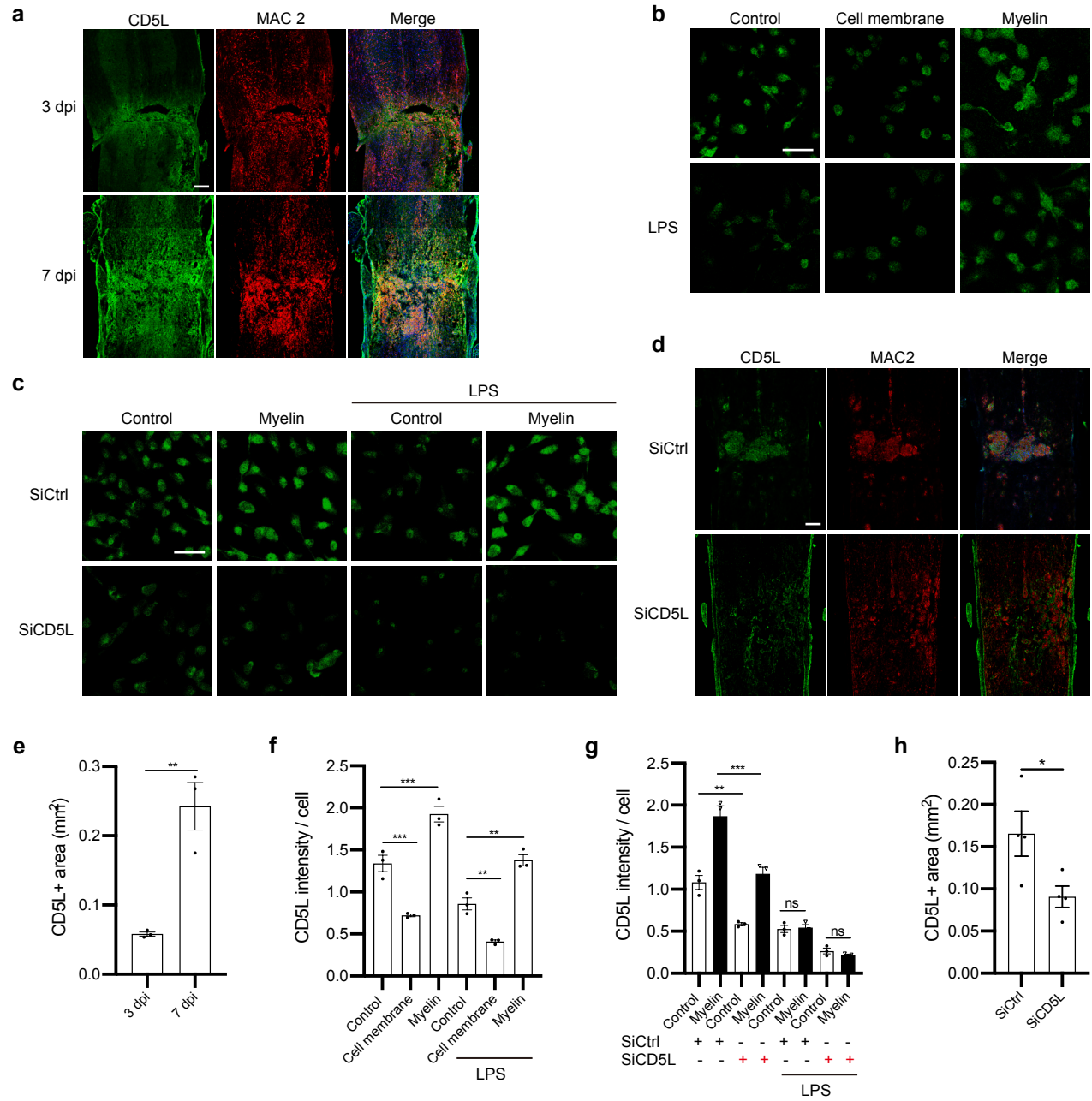

Supplement: Supplementary Materials — Fig. S1. Cholesterol crystals appear in spinal cord lesions. Fig. S2. Evaluation of interventions. Fig. S3. No deposition of cholesterol in uninjured sciatic nerves. Fig. S4. Limited cholesterol clearance reduces nerve fiber density. Fig. S5. Deposition and removal of cholesterol from macrophages in vitro. Fig. S6. Macrophages deposit in spinal cord lesions on account of myelin-derived lipids. Fig. S7. Myelin internalization promotes the expression of CD5L Table S1. List of primer sequences for real-time PCR. [file research.0135.f1.zip › Supplementary Figure 7.pdf]

**a**

LFB area

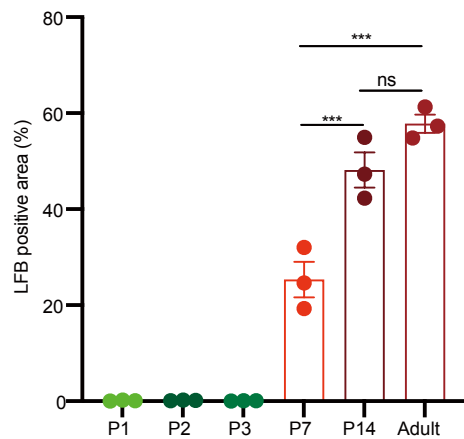**b**

MBP area

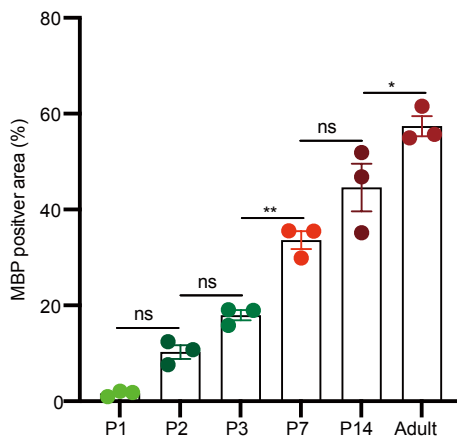**c**

IBA1

MAC2

Merge

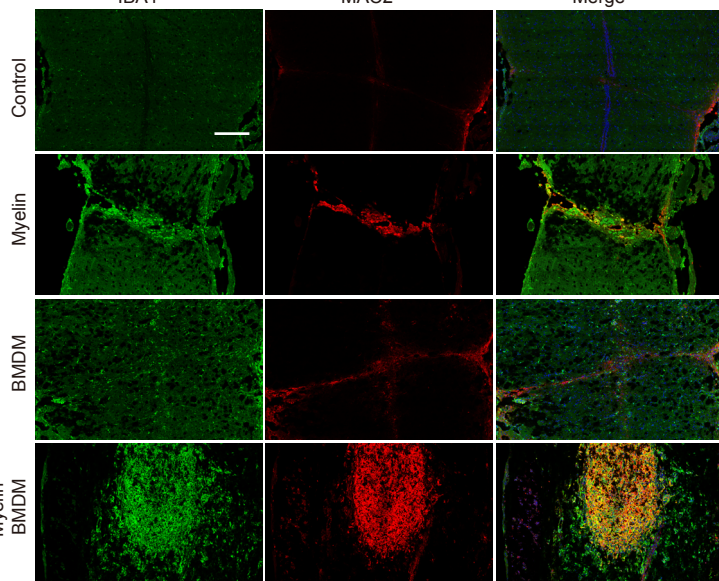**d**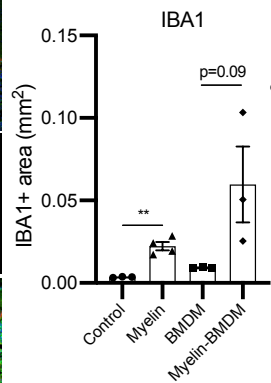**e**

MAC2

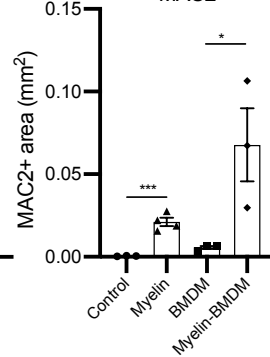

Supplement: Supplementary Materials — Fig. S1. Cholesterol crystals appear in spinal cord lesions. Fig. S2. Evaluation of interventions. Fig. S3. No deposition of cholesterol in uninjured sciatic nerves. Fig. S4. Limited cholesterol clearance reduces nerve fiber density. Fig. S5. Deposition and removal of cholesterol from macrophages in vitro. Fig. S6. Macrophages deposit in spinal cord lesions on account of myelin-derived lipids. Fig. S7. Myelin internalization promotes the expression of CD5L Table S1. List of primer sequences for real-time PCR. [file research.0135.f1.zip › Supplementary Figure 6.pdf]

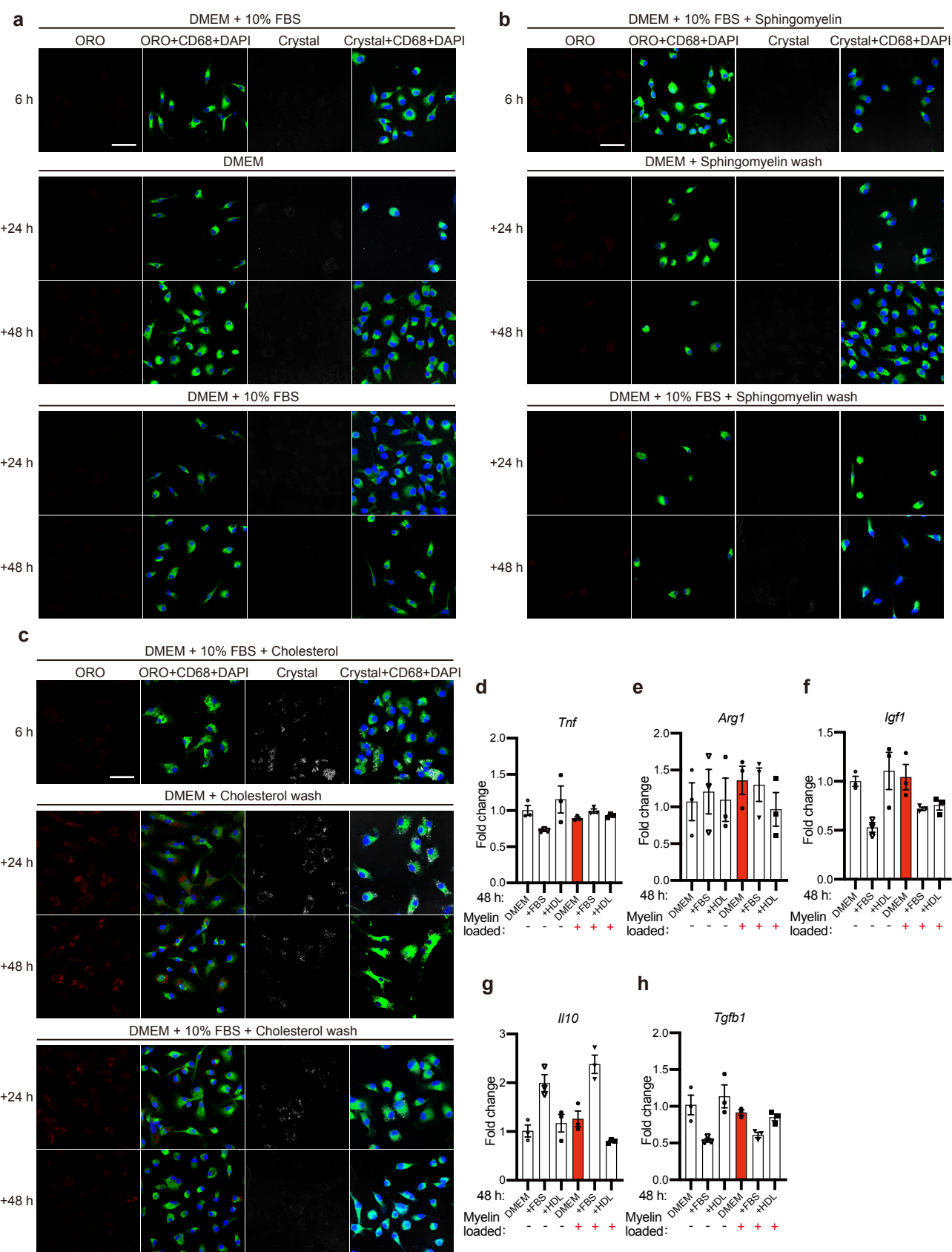

Supplement: Supplementary Materials — Fig. S1. Cholesterol crystals appear in spinal cord lesions. Fig. S2. Evaluation of interventions. Fig. S3. No deposition of cholesterol in uninjured sciatic nerves. Fig. S4. Limited cholesterol clearance reduces nerve fiber density. Fig. S5. Deposition and removal of cholesterol from macrophages in vitro. Fig. S6. Macrophages deposit in spinal cord lesions on account of myelin-derived lipids. Fig. S7. Myelin internalization promotes the expression of CD5L Table S1. List of primer sequences for real-time PCR. [file research.0135.f1.zip › Supplementary Figure 5.pdf]

**a**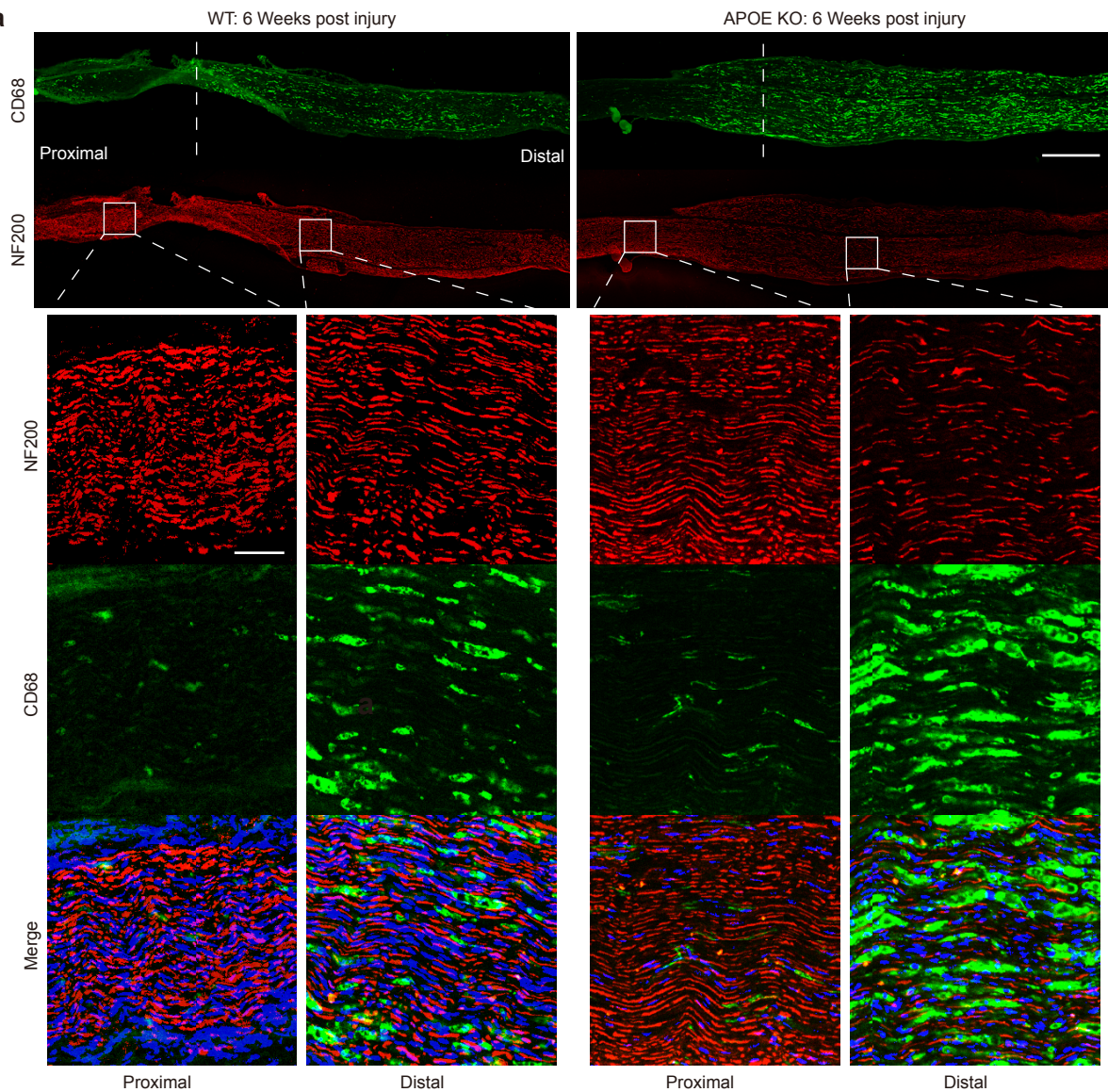**b**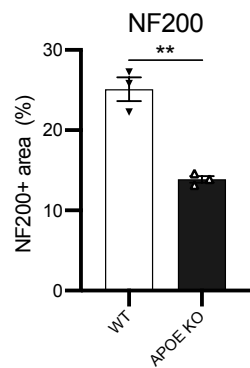**c**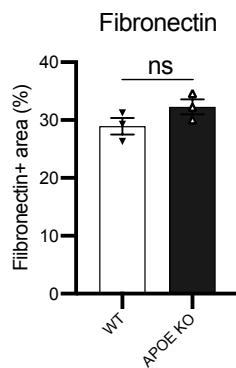**d**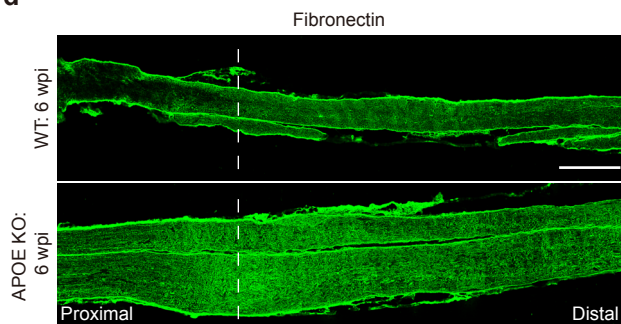

Supplement: Supplementary Materials — Fig. S1. Cholesterol crystals appear in spinal cord lesions. Fig. S2. Evaluation of interventions. Fig. S3. No deposition of cholesterol in uninjured sciatic nerves. Fig. S4. Limited cholesterol clearance reduces nerve fiber density. Fig. S5. Deposition and removal of cholesterol from macrophages in vitro. Fig. S6. Macrophages deposit in spinal cord lesions on account of myelin-derived lipids. Fig. S7. Myelin internalization promotes the expression of CD5L Table S1. List of primer sequences for real-time PCR. [file research.0135.f1.zip › Supplementary Figure 4.pdf]

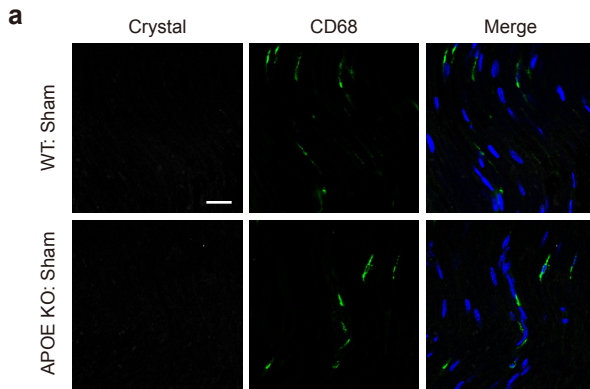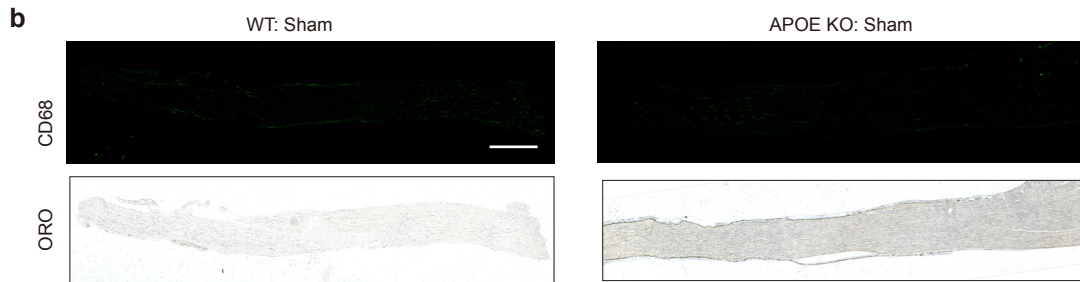

Supplement: Supplementary Materials — Fig. S1. Cholesterol crystals appear in spinal cord lesions. Fig. S2. Evaluation of interventions. Fig. S3. No deposition of cholesterol in uninjured sciatic nerves. Fig. S4. Limited cholesterol clearance reduces nerve fiber density. Fig. S5. Deposition and removal of cholesterol from macrophages in vitro. Fig. S6. Macrophages deposit in spinal cord lesions on account of myelin-derived lipids. Fig. S7. Myelin internalization promotes the expression of CD5L Table S1. List of primer sequences for real-time PCR. [file research.0135.f1.zip › Supplementary Figure 3.pdf]

**a**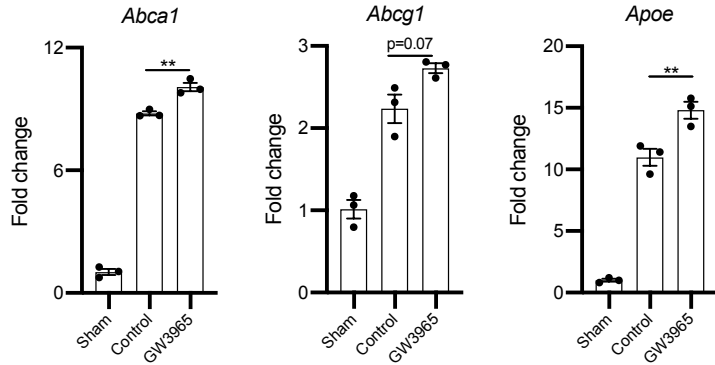**b**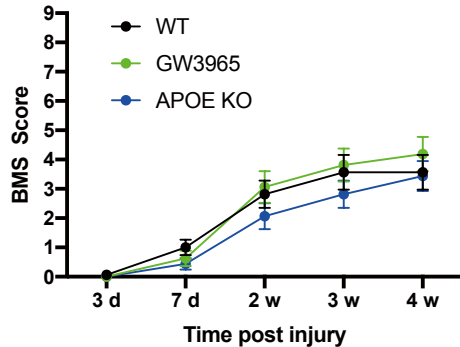

Supplement: Supplementary Materials — Fig. S1. Cholesterol crystals appear in spinal cord lesions. Fig. S2. Evaluation of interventions. Fig. S3. No deposition of cholesterol in uninjured sciatic nerves. Fig. S4. Limited cholesterol clearance reduces nerve fiber density. Fig. S5. Deposition and removal of cholesterol from macrophages in vitro. Fig. S6. Macrophages deposit in spinal cord lesions on account of myelin-derived lipids. Fig. S7. Myelin internalization promotes the expression of CD5L Table S1. List of primer sequences for real-time PCR. [file research.0135.f1.zip › Supplementary Figure 2.pdf]

**a**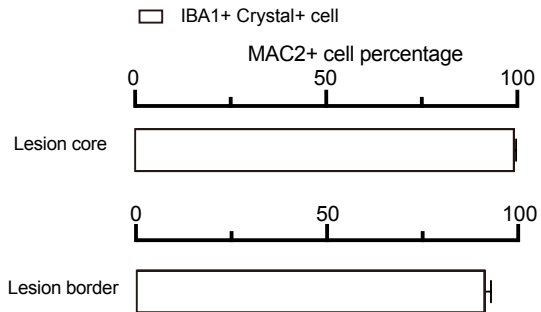**b**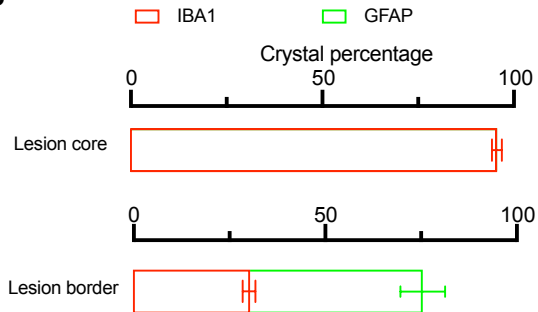**c**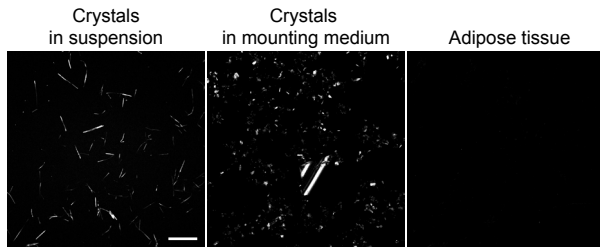**d**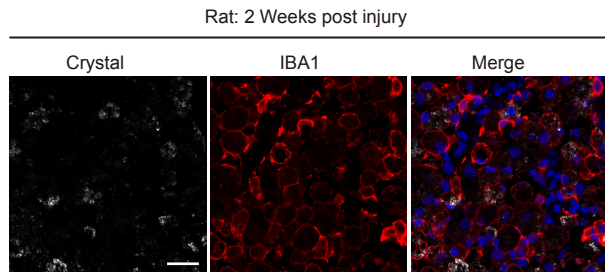

Supplement: Supplementary Materials — Fig. S1. Cholesterol crystals appear in spinal cord lesions. Fig. S2. Evaluation of interventions. Fig. S3. No deposition of cholesterol in uninjured sciatic nerves. Fig. S4. Limited cholesterol clearance reduces nerve fiber density. Fig. S5. Deposition and removal of cholesterol from macrophages in vitro. Fig. S6. Macrophages deposit in spinal cord lesions on account of myelin-derived lipids. Fig. S7. Myelin internalization promotes the expression of CD5L Table S1. List of primer sequences for real-time PCR. [file research.0135.f1.zip › Supplementary Figure 1.pdf]
